# Container volume may affect growth rates of ciliates and clearance rates of their microcrustacean predators in microcosm experiments

**DOI:** 10.1093/plankt/fbab017

**Published:** 2021-03-17

**Authors:** Thomas Weisse, Dunja Lukić, Xiaoteng Lu

**Affiliations:** University of Innsbruck, Research Department for Limnology, Mondseestr. 9, A-5310 Mondsee, Austria; University of Innsbruck, Research Department for Limnology, Mondseestr. 9, A-5310 Mondsee, Austria; University of Innsbruck, Research Department for Limnology, Mondseestr. 9, A-5310 Mondsee, Austria

**Keywords:** ciliates, volume effect, *Daphnia*, *Eudiaptomus*, *Cyclops*, growth and clearance rates

## Abstract

We studied the effect of volume in small containers (microcosms) on five common planktonic freshwater ciliates and three zooplankton species, namely *Daphnia* sp., the calanoid copepod *Eudiaptomus* sp., and the cyclopoid copepod *Cyclops* sp. We measured ciliate specific growth rates and their loss rates due to microcrustacean predation in short-term experiments. We hypothesized that container volume ranging from 10 to 200 mL would not affect the activity of our prey and predator species. We found that the response to volume was species-specific; growth rates of three ciliate species were sensitive to volume. However, the volume effect was not unequivocal because different timing of the microcosm experiments (block effects) may have caused random bias due to varying morphological and/or physiological conditions of the ciliates and their predators. For predator clearance rate, the volume effect was insignificant in the filter-feeding *Daphnia* and *Eudiaptomus* but was significant for the predatory copepod *Cyclops*, which was hampered in the smallest experimental containers. Total crustacean clearance rates averaged over all treatments appeared unaffected by predator species, while ciliate species significantly affected the results. Our growth and clearance rates are close to previous findings with the same or similar planktonic prey and predator species.

## INTRODUCTION

Choice of scale in experiments may affect results, but the effect of scaling has rarely been studied in experimental aquatic ecosystems. Laboratory experiments are widely used to investigate the effect of one or several environmental variables on the performance of an organism or a population. As a rule, the experimental area (for terrestrial organisms) or volume (for aquatic organisms) scales positively with the size of the organisms. For prokaryotic and eukaryotic microplankton organisms, the volume of the experimental containers (“microcosms”) usually ranges from 0.002 L to several L. In the literature, there is no clear distinction between the terms “microcosms” and “mesocosms.” Relative to the natural situation, microcosms represent a scale reduction of several orders of magnitude, while mesocosms represent a reduction of about two orders of magnitude or less (Lawler, 1998).

Microcosm experiments have been applied to test general ecological and evolutionary concepts with heterotrophic and phototrophic bacteria (Buckling *et al.*, 2000; Lenski, 2004; Brockhurst *et al.*, 2007), in phytoplankton ecology and physiology (reviewed by Nogueira *et al.*, 2014), in heterotrophic protists (reviewed by Altermatt *et al.*, 2015) and in various zooplankton species (e.g., Rothhaupt, 1997). For instance, for pragmatic reasons, in life-table experiments with rotifers investigating various life-history traits a higher population density (“crowding”) is reached by reducing the experimental volume (Gilbert, 2003). Experimental containers as small as 0.015–0.2 mL have been used to investigate demographic parameters and the mixing threshold, i.e. the population density necessary to induce production of males in parthenogenetic species (Walz, 1983, 1987; Carmona *et al.*, 2009; Cieplinski *et al.*, 2018, 2019). Similarly, Gause (1934) used test tubes of 10-mL volume in his classical competitive exclusion experiments with *Paramecium*.

A common concern in microcosms is that they are prone to numerous “bottle effects” that have been known for a long time (Pernthaler and Amann, 2005; Hammes *et al.*, 2010). Bottle effects are mainly related to experimental volume, or surface area-to-volume ratio (S/V), respectively, and experimental duration (Petersen *et al.*, 1999; Englund and Cooper, 2003). Surface area-to-volume ratios (S/V) generally decrease with increasing volume (Petersen *et al.*, 1999) but also depend on the shape of the containers. Walls of the microcosms are artifacts that can potentially alter light, heat and turbulent energy transfer, and often harbor an uncontrollable biofilm of attached organisms (Petersen *et al.*, 1999). However, surprisingly few studies investigated the effect of container volume on the experimental outcome in microcosm experiments (reviewed by Englund and Cooper, 2003; Altermatt *et al.*, 2015). The latter review on protist microcosms did not consider a potential effect of container size on the experimental results (Altermatt *et al.*, 2015). It appears that scaling has been largely ignored in the design of experimental aquatic ecosystems (Petersen *et al.*, 1999). To this end, we studied the volume effect in small containers ranging from 10 to 200 mL on five ciliate and three zooplankton species. Our hypothesis was that volume of the containers would not affect the activity of our prey and predator species.

Ciliate loss rates are primarily caused by predation of microcrustaceans, both in freshwater and in the oceans (Wickham, 1998; Adrian and Schneider-Olt, 1999; Burns and Schallenberg, 2001; Calbet and Saiz, 2005; Armengol *et al.*, 2017). In this study, we investigated ciliate grazing by three common freshwater microcrustaceans, i.e. a cladoceran (*Daphnia* sp.), a calanoid (*Eudiaptomus* sp.) and a cyclopoid (*Cyclops* sp.) copepod. *Daphnia* is a highly efficient filter feeder, ingesting particles ranging in size from bacteria to ciliates and small rotifers (Gilbert, 1988a, b; Wickham, 1998). *Eudiaptomus* feeds more selectively by actively grasping large particles (ambush feeding, Kiørboe, 2011a) and passively filtering small particles (Lampert and Muck, 1985; George and Hewitt, 1999; Gliwicz, 2003). *Cyclops* is a predatory species that is actively hunting its prey (Wickham, 1998; Gliwicz, 2003; Kiørboe, 2011a). We report that, overall, volume significantly affected the activity of the larger predatory microcrustaceans and may also, species-specifically, affect the activity of their ciliate prey.

## METHOD

### Study organisms

All heterotrophic species used in this study were isolated from mesotrophic Lake Mondsee (47°49′41.88”N, 13°22′46.56″E) located in the Austrian Salzkammergut lake district. Maximum water depth is 68 m; theoretical water renewal time is 1.82 years. Ciliates and microcrustaceans were sampled from the upper 30 m in spring and summer 2018, i.e. the ciliates and their predators had been kept in culture for up to half a year before the beginning of the experiments. The phototrophic flagellate *Cryptomonas* sp. strain 26/80, which served as food for the ciliates and *Daphnia*, was obtained from the Algenkultursammlung Göttingen (SAG). The ciliate species used in the experiments were the free-swimming peritrich *Vorticella natans* (41 × 36 μm), the scuticociliate *Histiobalantium bodamicum* (53 × 38 μm), the prostomatid *Urotricha* sp. (18 × 14 μm) and the choreotrich ciliates *Rimostrombidium caudatum* (59 × 47 μm) and *R. lacustris* (73 × 66 μm). Stem cultures were kept in modified Woods Hole medium (WMC, Guillard and Lorenzen 1972). The experimental containers were inoculated with ciliates from exponentially growing cultures.

*Daphnia* sp. was kept with *Cryptomonas* sp. as food. The calanoid copepod *Eudiaptomus* sp. was reared with a mixture of *Cryptomonas* sp. and the medium-sized ciliate *H. bodamicum.* For the cyclopoid copepod *Cyclops* sp., we used a mixture of *Cryptomonas* sp. and the ciliates *H. bodamicum, R. caudatum* and *Rimostrombididium lacustris.*

The experiments were conducted with female *Daphnia* and adult or late copepodite stages (C4–C6) of copepods, respectively. The species identity of the microcrustaceans used in the experiments was verified by Christian Jersabek (Lamprechtshausen, Austria). Most of the *Daphnia* specimens belonged to *Daphnia hyalina*; however, some individuals were hybrids of *D. hyalina* and *D. galeata* (*Daphnia* x *obscura*). The calanoid copepods were identified as *Eudiaptomus gracilis*. Adult cyclopoids belonged to the subspecies *Cyclops abyssorum prealpinus*. The taxonomic affiliation of some copepodites was not unequivocally clear; therefore, we refrain from reporting the results of the microcrustacean predation at the species level in the following.

### Experimental design

All experimental cultures were kept in sterile filtered and autoclaved water sampled from L. Mondsee at 30 m depth, which is below the euphotic zone. The experiments were conducted under a 14:10 h light:dark photoperiod at low light intensity (13–18 μmol photon m^−2^ s^−1^, Bergkemper *et al.*, 2018), which simulated the natural light cycle in spring through autumn at ~5–10 m depth in L. Mondsee (Bergkemper and Weisse, 2017 and own unpublished research). The experimental temperature was 15°C, which is close to the mean temperature in the epilimnion of the lake between May and October (Crosbie *et al.*, 2003; Dokulil and Teubner, 2012). The experimental duration was 24 h.

**Fig. 1:**
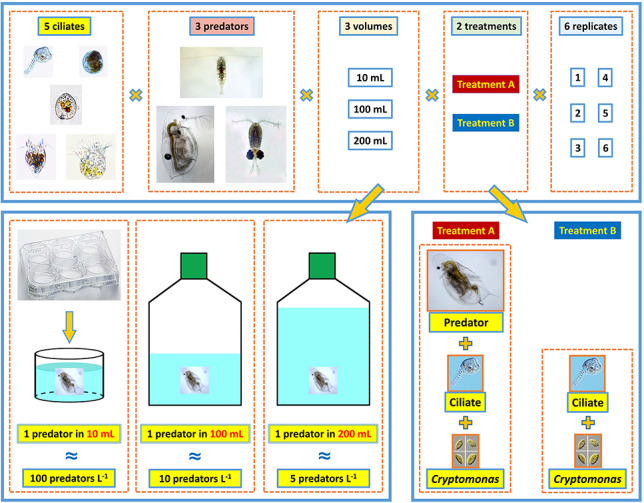
Experimental design of the microcosm experiments. Treatment A refers to microcrustacean predation, treatment B to ciliate growth rates.

We used five ciliate species and three crustacean predators at three volume levels (Fig. 1). To study the volume effect and mimic *in situ* predator abundances ranging from 5 to 100 ind. L^−1^, we used experimental volumes ranging from 10 to 200 mL. For the 10-mL experiments, 6-well culture plates (CytoOne®, STARLAB, Hamburg, Germany) received one predator each, corresponding to a theoretical predator abundance of 100 ind. L^−1^. To obtain lower predator abundance of 10 and 5 ind. L^−1^, cell culture flasks (CytoOne®) were used. Since one predator was added to each flask of 100-mL volume, the theoretical predator density was 10 ind. L^−1^; adding one microcrustacean to each 200-mL container yielded a predator density of 5 ind. L^−1^.

Each experiment consisted of two treatments (A, B) with six replicates each. Treatment A contained one predator species, one ciliate species and *Cryptomonas* sp.. Treatment B contained the same ciliate species and *Cryptomonas* sp., but no predators. Treatment A was used to calculate changes of ciliate abundance in the presence of predators, whereas treatment B was used to calculate changes of ciliate abundance (i.e. growth rates [*μ*], Eq. 1, below) in the absence of predators. Predator grazing impact can be derived from the relative difference in the change of cell numbers between treatments A and B (Eq. 2, below).

In total, the experiments comprised 5 ciliate species, 3 crustacean species, 3 volume levels and 2 treatments with 6 replicates each, yielding 540 experimental microcosms (Fig. 1). Since handling of this large number of experimental containers with the respective organisms was impossible in a short time, we conducted the whole experimental series at 28 different dates (blocks, see Supplementary Table SIII). Each block usually consisted of 24 microcosms. The combinations of ciliate and predators in each block were chosen according to the availability of ciliates and microcrustaceans meeting the criteria of exponentially growing ciliate cultures and adult or late copepodite crustaceans, respectively. The experiments were conducted from July to December 2018 and from August to September 2019.

Ciliates were added to each experimental microcosm at a target concentration of 1 cell mL^−1^. The food organism, *Cryptomonas* sp., was added to reach an experimental abundance of 15 000 cells mL^−1^, corresponding to ~0.5 mg C L^−1^ (Lu *et al.*, 2021). We acclimated those cultures for 24 h at the experimental conditions. If necessary, ciliates and their food were readjusted to the target concentrations before beginning the experiments. Microcrustaceans were kept at comparable moderate food concentrations of *Cryptomonas* sp. and prey ciliates (see above) during the acclimation phase.

### Abundance and size estimates

*Cryptomonas* sp. cells were counted and sized alive by an electronic particle counter (CASY® 1 Modell TTC, Weisse and Kirchhoff, 1997). Ciliates were fixed with Lugol’s solution and counted by inverted microscopy in counting chambers of 50 mL volume (Hydrobios, Kiel, Germany). Cell size of live ciliates grown at comparable food concentration as used in the experiments was measured from images taken by an imaging cytometer (FlowCam, Bergkemper and Weisse, 2018; Wirth *et al.*, 2019) and analyzed by an image analysis system (NIS elements D; Nikon CEE GmbH, Vienna, Austria) connected to an inverted microscope (Zeiss Axiovert 200).

*Daphnia* sp. was fixed with formalin-40% sucrose solution at the end of each experiment, and copepods were narcotized with filtered lake water amended with carbon dioxide before sizing. Lengths and widths of *Daphnia* sp. and the copepod species were measured at a magnification of 100× by the image analysis system reported above. Body length was measured from the anterior margin of head to the base of the posterior spine in *Daphnia* and from the anterior margin of head to posterior margin of urosome in copepods (i.e. caudal rami were not included in measurement; Rosen, 1981).

### Data acquisition and analysis

Ciliate specific growth rate (*μ*, d^−1^) was determined in treatment B assuming exponential growth according to the equation:

(1) \begin{equation*} \mu =\frac{\ln{N}_t-\ln{N}_0}{\left({t}_1-{t}_0\right)} \end{equation*}

where *N*_t_ and *N*_0_ are the final and initial population sizes, and *t*_0_ and *t*_1_ are the initial and final time in days.

Microcrustacean grazing rates (*g*, d^−1^) were calculated according to Frost (1972) and Weisse and Frahm (2002) as

(2) \begin{equation*} g=\frac{\ln \left({Cc}_t/ {Cc}_0\right)-\kern0.5em \ln \left({C}_t/{C}_0\right)}{\left({t}_1-{t}_0\right)} \end{equation*}

where *Cc_0_* and *Cc_t_* are the initial and final ciliate numbers in the controls (treatment B) and *C*_0_ and C*_t_* are the initial and final ciliate concentrations in the containers with microcrustaceans (treatment A). The grazing rate *g* divided by the number of microcrustacean predators (L^−1^) yielded the per capita grazing or clearance rate (L individual^−1^ d^−1^).

Statistical analyses were conducted with the software R (R Core Team, 2020), including the following packages: “stats,” “lmerTest,” “lme4” and effects for linear model (LM) and linear mixed-model analyses; “lmerTest” for analysis of variance (ANOVA) procedures and ANOVA-like output for random effects; “AICcmodavg” and “MuMIn” for model selection based on the Akaike information criterion (AIC); “graphics,” “ggplot2” and “beeswarm” for graphic output; “rstatix” for detecting outliers.

In several cases, calculated clearance rates were negative, which is unrealistic. Since ANOVA procedures are sensitive to outliers, negative clearance rates were removed from the analysis. We first analyzed the entire data set consisting of all growth rates and all positive grazing rates. We then identified further outliers both for growth and clearance rates using boxplots methods with the package “rstatix,” which are based upon the interquartile range (IQR) rule, i.e. the difference between means of the 75th and 25th percentiles (IQR = *Q*_3_ − *Q*_1_). Outliers were randomly distributed across the three volume levels. The resulting data set consisted of 262 (out of 270) values for growth rates and 237 values for clearance rates. The ANOVA analyses were diagnosed for violations of assumptions by using *Q*–*Q* plots and by plotting the residuals vs the fitted values, the standardized residuals vs the fitted values, and the residuals vs the leverage. The final ANOVA analyses conformed to the assumptions.

To account for volume and block effects, we used LM and linear mixed-effects models (LMEM) for growth and LM for clearance rate analyses (Tables I and II). Growth rate of each ciliate species was analyzed with volume as fixed factor and “block” as random factor. Because we compared LMEM and LM models, maximum likelihood (ML) was used for model fitting in LMEM. Clearance rate was analyzed at each predator level, with volume and ciliate species as fixed factors (with and without interaction). Since each specific ciliate × predator × volume combination was only run in one block and not distributed across several blocks, we could not test for block effects on clearance rates.

**Table I:** Model results for ciliate growth rates (all data without outliers, n = 262). Best fits indicated by AIC scores in red, significant effects in bold face. LMEM - linear mixed-effects models: Growth.rate ~ Volume + (1|Block); LM - linear model: Growth.rate ~ Volume; the volume column reports F values and corresponding p values in brackets. The block column contains LRT (Likelihood Ratio Test) statistics and corresponding p values in parentheses

Ciliate	LMEM				LM	
	AIC	Variance proportion	Volume	Block	AIC	Volume
*Urotricha sp.*	-141.922	0.500	**17.782 (<0.001)**	**15.122 (<0.001)**	-129.234	**30.910 (<0.001)**
*Vorticella natans*	-28.748	0.429	1.207 (0.345)	**12.371 (<0.001)**	-18.841	2.922 (0.063)
*Histiobalantium bodamicum*	-30.057	0.412	**14.635 (<0.001)**	**7.100 (0.007)**	-25.443	**27.063 (<0.001)**
*Rimostrombidium caudatum*	-9.928	0.627	**0.275 (0.765)**	**31.757 (<0.001)**	19.395	1.391 (0.258)
*Rimostrombidium lacustris*	3.835	0.500	**6.648 (0.005)**	**20.197 (<0.001)**	21.599	2.121 (0.130)

**Table II:** Model results for microcrustacean clearance rates (all data without outliers, n = 237). Models with best fits indicated by AIC scores in red, significant effects in bold face. LM - linear model: clearance rate ~ ciliate + volume; LM.int - linear model with interaction: clearance rate ~ ciliate × volume. the volume, ciliate and interaction columns report F values and corresponding p values in parentheses

Predator	LM			LM.int			
	AIC	Volume	Ciliate	AIC	Volume	Ciliate	Interaction
*Daphnia*	-487.662	1.807 (0.172)	**9.626 (<0.001)**	-481.021	1.983 (0.146)	**10.564 (<0.001)**	1.840 (0.087)
*Eudiaptomus*	-545.160	0.681 (0.509)	**19.306 (<0.001)**	-535.732	0.710 (0.495)	**20.141 (<0.001)**	1.416 (0.205)
*Cyclops*	-433.491	**11.648 (<0.001)**	1.420 (0.236)	-463.782	**20.599 (<0.001)**	2.512 (0.051)	**7.723 (<0.001)**

Experimental results reported in the text represent mean values *+* standard deviation (SD). Results were considered significant if *P* was ≤0.05.

## RESULTS

### Model selection and results

Removing outliers from the data sets did not affect the conclusions derived from the model results (Table I and Supplementary Table SI). Provided that the number of observations is the same, the model with the lowest AIC represents the model with the best-fit, and models with differences (Δ, delta) in AIC scores > 2 are usually considered different (Burnham and Anderson, 2004; Bolker, 2008). Based on this criterion, LMEM yielded the best fit for growth rates of all ciliates. The volume effect was only insignificant in *Vorticella natans* (*P* = 0.345; Table I) and *Rimostrombidium caudatum* (*P* = 0.765; Table I).

LM yielded significant results for all predators; clearance rates of *Daphnia* and *Eudiaptomus* were better fitted without volume × ciliate interaction, *Cyclops* with this interaction (Table II). Similar to ciliate growth rates, removing outliers did not affect the conclusions derived from the model results (cf. Table II and Supplementary Table SII). According to the model outcome, the response to the container volume was species-specific. The volume effect was insignificant in the filter-feeding *Daphnia* (*P* = 0.172) and *Eudiaptomus* (*P* = 0.509), but significant for the predatory copepod *Cyclops* (*P* < 0.001).

### Ciliate specific growth rates

Mean ciliate growth rates ranged from negative values recorded in one experiment with *Vorticella natans* and three experiments with *Histiobalantium bodamicum* to 1.01 d^−1^ measured for *R. lacustris* in the “*Cyclops*” treatment in the largest container. Lowest mean growth rates across all treatments were measured for *V. natans* (0.214 *+* 0.198 d^−1^), highest growth rates for *R. lacustris* (0.607 *+* 0.286 d^−1^). The median growth rate for all five species was 0.35 d^−1^. Details on specific growth rates of the study ciliates will be reported elsewhere (Lu *et al*., in preparation).

**Fig. 2:**
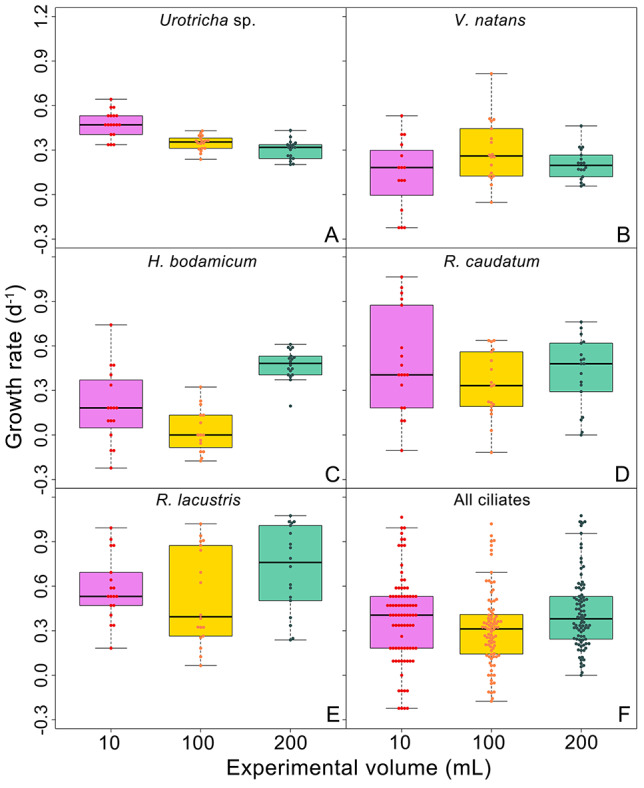
Box and bee swarm plots of ciliate growth rates in relation to container volume. For each ciliate species, the lower boundary of the box indicates the 25^th^ percentile, the solid line within the box marks the median, and the upper boundary of the box indicates the 75^th^ percentile. Whiskers (error bars) above and below the box indicate the 90^th^ and 10^th^ percentiles.

The boxplots (Fig. 2) suggest that ciliate growth rates differed between the experimental volumes for *Urotricha* sp. (Fig. 2A), *H. bodamicum* (Fig. 2C) and *R. lacustris* (Fig. 2E), which was confirmed by the statistical analyses (Table I). In contrast, *V. natans* (Fig. 2B) and *R. caudatum* (Fig. 2D) were insensitive to the experimental volume. Considering block effect as random factor (LMEM) improved the model fit in each case, relative to LM models without random effects (Table I).

### Microcrustacean clearance rates

**Fig. 3:**
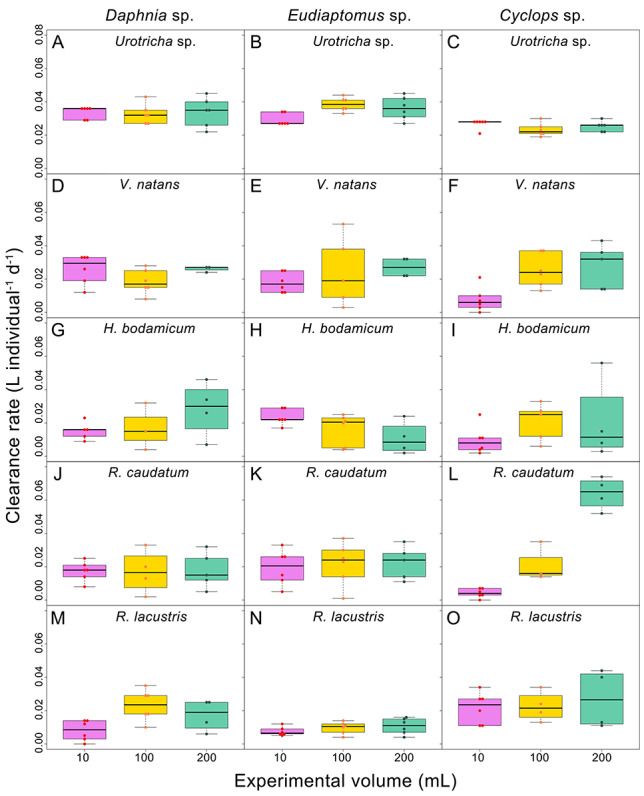
Clearance rates of the three microcrustacean species on the five ciliate species in relation to container volume. Same legend as in Figure 2.

Predation by microcrustaceans (Fig. 3) was less variable than ciliate growth rates. An exception was *Cyclops* sp. feeding on *Rimostrombidium caudatum* (Fig. 3L). The mean ciliate-specific clearance rate of this predators varied twofold, from 0.016 *+* 0.010 L individual^−1^ d^−1^ in *R. lacustris* to 0.031 *+* 0.007 L individual^−1^ d^−1^ in *Urotricha* sp.. Averaged over all treatments A, the clearance rate of the microcrustaceans was 0.022 *+* 0.010 L individual^−1^ d^−1^ and was unaffected by predator species. In contrast, volume, ciliate species and all interactions significantly affected the results (Table II). Details on ciliate-specific clearance rates and their ecological consequences will be discussed in a related article (Lu and Weisse, in preparation).

Results from LM analyses indicated that the volume effect and volume × ciliate interaction was only significant in one species, *Cyclops* sp. (Table II). The mean clearance of this predatory copepod was strongly reduced in the smallest containers (10 mL, average 0.014 *+* 0.011 L individual^−1^ d^−1^), relative to the largest enclosures (200 mL, average 0.032 *+* 0.020 L individual^−1^ d^−1^) used in this study (Fig. 3F, I, L).

## DISCUSSION

### Main results

The response to volume was species-specific for the study ciliates and their predators. Growth rates of three ciliate species appeared affected by the experimental volume; only *Vorticella natans* and *R. caudatum* were insensitive to the volume of the microcosms. However, the volume effect was not unequivocal because different timing of the microcosm experiments (block effects) caused random bias, probably due to varying morphological and/or physiological conditions of the ciliates and their predators (discussed below). For predator clearance rate, the volume effect was not significant in the filter-feeding *Daphnia* and *Eudiaptomus* but was significant for the predatory copepod *Cyclops*, which was hampered in the smallest experimental containers.

### Advantages and limitations of small microcosms

Previous studies reported no significant or only minor volumetric effects on the experimental outcome in aquatic microcosms. Typical examples were provided by Hammes *et al.* (2010) and Nogueira *et al.* (2014), who found no evidence of a volumetric bottle effect on bacterial and phytoplankton growth rates, respectively. Similarly, Carrick *et al.* (1992) found generally good agreement between *in vitro* and *in situ* growth rate estimates for flagellates and ciliates in Lake Michigan; however, these authors also noted that sensitivity to containment can be species-specific and reported that the ciliate *Strombidium* sp. was adversely affected by incubation in 4-L bottles.

The pros and cons of microcosm experiments for investigating ecological processes and ecosystem properties have been controversially discussed in the literature (Carpenter, 1996; Lawler, 1998; Hammes *et al.*, 2010; Altermatt *et al.*, 2015). Small microcosms (≤1 L) allow using more replicates per treatment and more treatments than larger containers (Petersen *et al.*, 1999). On the other hand, wall effects decrease with enclosure size (Englund and Cooper, 2003). Some ecological processes such as phytoplankton growth rates may be better controlled and manipulated in smaller than in larger enclosures. In contrast, investigations of complex species interactions inherent in multiple trophic level experiments including large predators require larger enclosures (Englund and Cooper, 2003). Similarly, the effect of physical processes such as (large-scale) turbulence or temperature gradients can only be studied in relatively large (several hundred to thousands of m^3^) mesocosms.

Only a few studies reported large negative “bottle effects”; testing bottle sizes ranging from 30 mL to 3.8 L, Gieskes *et al.* (1979) cautioned that the traditional ^14^C methods for measuring primary production in small bottles (≤250 mL) would grossly underestimate primary production in oceanic water. These authors concluded that the adverse effect may have been caused by increased mortality and limited nutrient regeneration in the small bottles. Wynn and Paradise (2001) found adverse effects of microcosm scaling on the growth and survival of larval stages of the mosquito, *Culex pipiens*. Microcosms with more vertical surface area/volume produced larger mosquitoes, probably because more food has been available during the experiments since mosquitoes browse on walls and other substrates for food.

Importantly, experimental results typically are influenced by more than one scale-dependent process (Englund and Cooper, 2003). An inherent assumption of the present study was that growth rates of prey would be independent of grazer activity. However, grazing by predators may stimulate growth rates of prey due to the release of nutrients and/or enzymes (reviewed by Gliwicz, 2003). If predator activity would be impeded by physical constraints or enhanced because predators trap prey in corners or predators and prey aggregate along walls (Bergström and Englund, 2002), this may affect prey growth rates. The fact that the predation rate by *Cyclops* sp. was significantly lower in the small wells (10 mL) than in the larger flasks (200 mL) suggests that this predatory species is sensitive to containment, and this may affect the ciliate prey. We assume that the effect of containment was linked to the reduced small-scale turbulence in the wells, relative to our larger containers. Since the seminal paper by Rothschild and Osborn (1988), it is known that microscale turbulence generally increases planktonic predator–prey contact rates. Turbulence is most important for meso-sized (mm to cm) predators, in particular for ambush feeding copepods such as many cyclopoids (Kiørboe and Saiz, 1995). For filter-feeding copepods and cladocerans, turbulence is of minor importance (reviewed by Kiørboe, 2011b). In agreement with the theoretical considerations, the filter feeders *Daphnia* sp. and *Eudiaptomus* sp. were unaffected by container size. As noted above, *Eudiaptomus* is also able to switch its feeding mode to ambush feeding, actively attacking its prey (Gliwicz, 2003; Kiørboe, 2011a, b; Kunzmann *et al.*, 2019). More research with microcrustaceans is needed to test if the effect of containment, irrespective of the effect of turbulence, depends on their different feeding modes.

### Block effects and model comparisons – limitations of the statistical analyses

Due to practical constraints, not all experiments could be performed simultaneously. Therefore, we split the whole experiment series into 28 dates (blocks). In our LMEM analyses, blocks were treated as a random factor, because combinations of ciliates and predators in each block were chosen randomly according to the availability of exponentially growing ciliates and adult or late copepodite crustaceans. Usually, we conducted two experiments in each block (i.e. on the same date), but the two experiments in one block did not always belong to the same “predator–prey” experimental series. For example, on 21–22 August 2018 (block 9, Supplementary Table SIII), we performed two experiments in parallel: (i) *Daphnia* feeding on *Vorticella* at 200 mL and (ii) *Daphnia* feeding on *R. caudatum* at 100 mL. The period between different blocks of one predator–prey experimental series ranged from 0 to 75 d; e.g. in the experiments with *Eudiaptomus* feeding on *Vorticella*, the experiments at 10, 100 and 200 mL volume were performed on 14–15 August 2018 (block 10), 11–12 September 2018 (block 11) and 30–31 October 2018 (block 13), respectively.

The morphological and/or physiological conditions of the ciliates and their predators may have varied from block to block. For ciliate growth rates, including blocks as a random factor (LMEM) improved the model fit in each case, relative to LM models (Table I). However, a clear relationship between the average growth rate and volume was only obvious in one species. Counterintuitively, growth rates of *Urotricha* sp. were inversely related to container size (Fig. 2A).

### Scaling

The microcrustacean species that we used were approximately 20–50× larger in body size (longest linear body dimension without attachments) than the ciliates. Therefore, constraints imposed by the physical dimensions of the experimental containers on the activity of the study species appear more likely for zooplankton than ciliates. Large individuals of the largest zooplankton species investigated, *Daphnia* sp., were approximately 2 mm in size. The physical dimensions of our smallest experimental containers, the 6-well culture plates, were 35 × 13 mm (width and depth at an experimental volume of 10 mL). Therefore, the dimensions of the container were only 7–18 × larger than the body size of *Daphnia* sp.. Confinement in these small containers did not affect the filtration and grazing activity of the cladoceran. Among the copepod species, the calanoid *Eudiaptomus* sp. was, on average, slightly larger than the cyclopoid *Cyclops* sp. (Lu and Weisse, in preparation). As discussed above, the different feeding modes of the microcrustaceans may have been more important than their body size regarding their sensitivity to confinement.

Similar clearance rates as we found have been reported earlier for copepods and *Daphnia* from differently sized enclosures (up to 10 L volume) using different experimental methods (Wiackowski *et al.*, 1994; Jack and Gilbert, 1997; Wickham, 1998; Adrian and Schneider-Olt, 1999; Agasild *et al.*, 2012; Armengol *et al.*, 2017). Using literature data, Jack and Gilbert (1997) listed typical clearance rates of 25 mL predator^−1^ d^−1^ for *Daphnia* and *Bosmina* and 30 mL predator^−1^ d^−1^ for calanoid copepods and *Cyclops abyssorum*.

For ciliates, even the smallest dimension of our containers (i.e. depth) was ~260× larger than ciliate size, rendering effects of containment less likely than for larger organisms. In support of this assumption, the highest ciliate specific growth rates reported in this study are close to maximum growth rates reported previously for the respective species *in vitro* (Müller and Geller, 1993; Müller and Weisse, 1994; Müller and Schlegel, 1999; Pedersen and Hansen, 2003; Weisse and Rammer, 2006; Lu and Weisse, in preparation) and *in situ* (Müller and Weisse, 1994; Müller *et al.*, 1991; Carrick *et al.*, 1992; Weisse, 2006) at comparable temperature (15°C). Apparently, the moderately high *Cryptomonas* sp. food concentration (~0.5 mg C L^−1^) that we used provided satiating conditions for (most of) the study species. Several of these previous studies were conducted as numerical response experiments in similar-sized microcosms as used in the present study.

To reduce potential wall effects, we used a relatively short experimental duration of 1 day. This period is close to the generation time of the ciliates and allows quantification of growth and loss rates per day.

## CONCLUSION

Volume affected the growth rates of three of the five studied ciliate species, but different timing of the experiments caused random effects that were likely related to the physiological conditions of the ciliates.

Among the three predators, the volume effect was only significant for the cyclopoid copepod *Cyclops*. For this species, a minimum experimental volume of 100–200 mL is required to yield unbiased results.

Some factors such as turbulence, parasitism and larger predators that were not considered in our experimental *in vitro*-design may affect ciliate growth and grazing loss rates *in situ*. Therefore, upscaling the laboratory work with microcosms to large-scale natural systems is problematic (Weisse *et al.*, 2016).

However, the evidence from the *in-situ* studies cited above suggests that those effects were minor in the present work, relative to bottom-up control via resources and top-down control by predation. We infer that at least the larger (100 and 200 mL) of the small microcosms that we used yielded realistic estimates of growth and grazing loss rates of the ciliates *in situ*. Accordingly, our results may be used to grossly calculate ciliate growth rates and their top-down control by microcrustaceans in lakes.

## DATA ARCHIVING

The data used to support the findings of this study are available from the corresponding author upon request.

## Supplementary Material

Supplementary_Tables_1-3_fbab017
