# Supplementary material for: Container volume may affect growth rates of ciliates and clearance rates of their microcrustacean predators in microcosm experiments
Source: J Plankton Res. 2021 Mar 17;43(2):288–99. doi: 10.1093/plankt/fbab017 (PMC8009685; doi:10.1093/plankt/fbab017)
Supplement: Supplementary_Tables_1-3_fbab017 [file supplementary_tables_1-3_fbab017.docx]

**SUPPLEMENTARY DATA**

Supplementary Table S1. Model results for ciliate growth rates (all data, n=270). Best fits indicated by AIC scores in red, significant effects in bold face. LMEM - linear mixed model: Growth.rate ~ Volume + (1|Block); LM - linear model: Growth.rate ~ Volume; LMEM.int.all - linear mixed model with interaction for all ciliates: Growth rate ~ Ciliate × Volume + (1 | Block). The volume column reports F values and corresponding p values in brackets. The block column contains LRT values and corresponding p value in parentheses.

| **Ciliate** | **LMEM** | | | | **LM** | |
| --- | --- | --- | --- | --- | --- | --- |
|  | **AIC** | **Variance proportion** | **Volume** | **(1\|Block)** | **AIC** | **Volume** |
| *Urotricha sp.* | -141.922 | 0.500 | **17.782 (<0.001)** | **15.122 (<0.001)** | -129.234 | **30.910 (<0.001)** |
| *Vorticella natans* | 0.222 | 0.245 | 2.834 (0.111) | **5.579 (0.018)** | 3.367 | **6.000 (0.005)** |
| *Histiobalantium bodamicum* | 9.190 | 0.121 | **14.007 (<0.001)** | 1.104 (0.293) | 7.860 | **21.180 (<0.001)** |
| *Rimostrombidium caudatum* | -9.928 | 0.627 | 0.275 (0.765) | **31.757 (<0.001)** | 19.395 | 1.391 (0.258) |
| *Rimostrombidium lacustris* | 3.835 | 0.500 | **6.648 (0.005)** | **20.197 (<0.001)** | 21.599 | 2.121 (0.130) |

Supplementary Table S2. Model results for microcrustacean clearance rates (all positive data, n=240). Models with best fits indicated by AIC scores in red, significant effects in bold face. LM - linear model: Clearance rate ~ Ciliate + Volume; LM.int - linear model with interaction: Clearance rate ~ Ciliate × Volume. Volume, Ciliate and Interaction columns contain F values and corresponding p values in parentheses.

| **Predator** | **LM** | | | **LM.int** | | | |
| --- | --- | --- | --- | --- | --- | --- | --- |
|  | **AIC** | **Volume** | **Ciliate** | **AIC** | **Volume** | **Ciliate** | **Interaction** |
| *Daphnia* | -476.599 | 1.655 (0.198) | **6.929 (<0.001)** | -468.503 | 1.778 (0.177) | **7.443 (<0.001)** | 1.649 (0.129) |
| *Eudiaptomus* | -545.160 | 0.681 (0.509) | **19.306 (<0.001)** | -535.732 | 0.710 (0.495) | **20.141 (<0.001)** | 1.416 (0.205) |
| *Cyclops* | -432.867 | **13.899 (<0.001)** | 0.904 (0.466) | -448.017 | **19.953 (<0.001)** | 1.298 (0.280) | **4.920 (<0.001)** |

Supplementary Table S3. Details of the experimental timing in 28 blocks.

| **Group** | **Volume** | **Block** | **Date** | **Ciliate species** | **Predator** |
| --- | --- | --- | --- | --- | --- |
| A1 | 10mL | 1 | 20190903-4 | *Urotricha* | *Daphnia* |
| A2 | 10mL | 1 | 20190903-4 | *Urotricha* | *Daphnia* |
| A3 | 10mL | 1 | 20190903-4 | *Urotricha* | *Daphnia* |
| A4 | 10mL | 1 | 20190903-4 | *Urotricha* | *Daphnia* |
| A5 | 10mL | 1 | 20190903-4 | *Urotricha* | *Daphnia* |
| A6 | 10mL | 1 | 20190903-4 | *Urotricha* | *Daphnia* |
| A1 | 100mL | 2 | 20190911-12 | *Urotricha* | *Daphnia* |
| A2 | 100mL | 2 | 20190911-12 | *Urotricha* | *Daphnia* |
| A3 | 100mL | 2 | 20190911-12 | *Urotricha* | *Daphnia* |
| A4 | 100mL | 2 | 20190911-12 | *Urotricha* | *Daphnia* |
| A5 | 100mL | 2 | 20190911-12 | *Urotricha* | *Daphnia* |
| A6 | 100mL | 2 | 20190911-12 | *Urotricha* | *Daphnia* |
| A1 | 200mL | 2 | 20190911-12 | *Urotricha* | *Daphnia* |
| A2 | 200mL | 2 | 20190911-12 | *Urotricha* | *Daphnia* |
| A3 | 200mL | 2 | 20190911-12 | *Urotricha* | *Daphnia* |
| A4 | 200mL | 2 | 20190911-12 | *Urotricha* | *Daphnia* |
| A5 | 200mL | 2 | 20190911-12 | *Urotricha* | *Daphnia* |
| A6 | 200mL | 2 | 20190911-12 | *Urotricha* | *Daphnia* |
| A1 | 10mL | 3 | 20190808-09 | *Urotricha* | *Eudiaptomus* |
| A2 | 10mL | 3 | 20190808-09 | *Urotricha* | *Eudiaptomus* |
| A3 | 10mL | 3 | 20190808-09 | *Urotricha* | *Eudiaptomus* |
| A4 | 10mL | 3 | 20190808-09 | *Urotricha* | *Eudiaptomus* |
| A5 | 10mL | 3 | 20190808-09 | *Urotricha* | *Eudiaptomus* |
| A6 | 10mL | 3 | 20190808-09 | *Urotricha* | *Eudiaptomus* |
| A1 | 100mL | 3 | 20190808-09 | *Urotricha* | *Eudiaptomus* |
| A2 | 100mL | 3 | 20190808-09 | *Urotricha* | *Eudiaptomus* |
| A3 | 100mL | 3 | 20190808-09 | *Urotricha* | *Eudiaptomus* |
| A4 | 100mL | 3 | 20190808-09 | *Urotricha* | *Eudiaptomus* |
| A5 | 100mL | 3 | 20190808-09 | *Urotricha* | *Eudiaptomus* |
| A6 | 100mL | 3 | 20190808-09 | *Urotricha* | *Eudiaptomus* |
| A1 | 200mL | 4 | 20190815-16 | *Urotricha* | *Eudiaptomus* |
| A2 | 200mL | 4 | 20190815-16 | *Urotricha* | *Eudiaptomus* |
| A3 | 200mL | 4 | 20190815-16 | *Urotricha* | *Eudiaptomus* |
| A4 | 200mL | 4 | 20190815-16 | *Urotricha* | *Eudiaptomus* |
| A5 | 200mL | 4 | 20190815-16 | *Urotricha* | *Eudiaptomus* |
| A6 | 200mL | 4 | 20190815-16 | *Urotricha* | *Eudiaptomus* |
| A1 | 10mL | 4 | 20190815-16 | *Urotricha* | *Cyclops* |
| A2 | 10mL | 4 | 20190815-16 | *Urotricha* | *Cyclops* |
| A3 | 10mL | 4 | 20190815-16 | *Urotricha* | *Cyclops* |
| A4 | 10mL | 4 | 20190815-16 | *Urotricha* | *Cyclops* |
| A5 | 10mL | 4 | 20190815-16 | *Urotricha* | *Cyclops* |
| A6 | 10mL | 4 | 20190815-16 | *Urotricha* | *Cyclops* |
| A1 | 100mL | 5 | 20190827-28 | *Urotricha* | *Cyclops* |
| A2 | 100mL | 5 | 20190827-28 | *Urotricha* | *Cyclops* |
| A3 | 100mL | 5 | 20190827-28 | *Urotricha* | *Cyclops* |
| A4 | 100mL | 5 | 20190827-28 | *Urotricha* | *Cyclops* |
| A5 | 100mL | 5 | 20190827-28 | *Urotricha* | *Cyclops* |
| A6 | 100mL | 5 | 20190827-28 | *Urotricha* | *Cyclops* |
| A1 | 200mL | 5 | 20190827-28 | *Urotricha* | *Cyclops* |
| A2 | 200mL | 5 | 20190827-28 | *Urotricha* | *Cyclops* |
| A3 | 200mL | 5 | 20190827-28 | *Urotricha* | *Cyclops* |
| A4 | 200mL | 5 | 20190827-28 | *Urotricha* | *Cyclops* |
| A5 | 200mL | 5 | 20190827-28 | *Urotricha* | *Cyclops* |
| A6 | 200mL | 5 | 20190827-28 | *Urotricha* | *Cyclops* |
| A1 | 10mL | 6 | 20180704-05 | *Vorticella* | *Daphnia* |
| A2 | 10mL | 6 | 20180704-05 | *Vorticella* | *Daphnia* |
| A3 | 10mL | 6 | 20180704-05 | *Vorticella* | *Daphnia* |
| A4 | 10mL | 6 | 20180704-05 | *Vorticella* | *Daphnia* |
| A5 | 10mL | 6 | 20180704-05 | *Vorticella* | *Daphnia* |
| A6 | 10mL | 6 | 20180704-05 | *Vorticella* | *Daphnia* |
| A1 | 100mL | 7 | 20180712-13 | *Vorticella* | *Daphnia* |
| A2 | 100mL | 7 | 20180712-13 | *Vorticella* | *Daphnia* |
| A3 | 100mL | 7 | 20180712-13 | *Vorticella* | *Daphnia* |
| A4 | 100mL | 7 | 20180712-13 | *Vorticella* | *Daphnia* |
| A5 | 100mL | 7 | 20180712-13 | *Vorticella* | *Daphnia* |
| A6 | 100mL | 7 | 20180712-13 | *Vorticella* | *Daphnia* |
| A1 | 10mL | 8 | 20180718-19 | *R.lacustris* | *Daphnia* |
| A2 | 10mL | 8 | 20180718-19 | *R.lacustris* | *Daphnia* |
| A3 | 10mL | 8 | 20180718-19 | *R.lacustris* | *Daphnia* |
| A4 | 10mL | 8 | 20180718-19 | *R.lacustris* | *Daphnia* |
| A5 | 10mL | 8 | 20180718-19 | *R.lacustris* | *Daphnia* |
| A6 | 10mL | 8 | 20180718-19 | *R.lacustris* | *Daphnia* |
| A1 | 200mL | 9 | 20180821-22 | *Vorticella* | *Daphnia* |
| A2 | 200mL | 9 | 20180821-22 | *Vorticella* | *Daphnia* |
| A3 | 200mL | 9 | 20180821-22 | *Vorticella* | *Daphnia* |
| A4 | 200mL | 9 | 20180821-22 | *Vorticella* | *Daphnia* |
| A5 | 200mL | 9 | 20180821-22 | *Vorticella* | *Daphnia* |
| A6 | 200mL | 9 | 20180821-22 | *Vorticella* | *Daphnia* |
| A1 | 100mL | 9 | 20180821-22 | *R.caudatum* | *Daphnia* |
| A2 | 100mL | 9 | 20180821-22 | *R.caudatum* | *Daphnia* |
| A3 | 100mL | 9 | 20180821-22 | *R.caudatum* | *Daphnia* |
| A4 | 100mL | 9 | 20180821-22 | *R.caudatum* | *Daphnia* |
| A5 | 100mL | 9 | 20180821-22 | *R.caudatum* | *Daphnia* |
| A6 | 100mL | 9 | 20180821-22 | *R.caudatum* | *Daphnia* |
| A1 | 10mL | 10 | 20180814-15 | *Vorticella* | *Eudiaptomus* |
| A2 | 10mL | 10 | 20180814-15 | *Vorticella* | *Eudiaptomus* |
| A3 | 10mL | 10 | 20180814-15 | *Vorticella* | *Eudiaptomus* |
| A4 | 10mL | 10 | 20180814-15 | *Vorticella* | *Eudiaptomus* |
| A5 | 10mL | 10 | 20180814-15 | *Vorticella* | *Eudiaptomus* |
| A6 | 10mL | 10 | 20180814-15 | *Vorticella* | *Eudiaptomus* |
| A1 | 10mL | 10 | 20180814-15 | *R.caudatum* | *Daphnia* |
| A2 | 10mL | 10 | 20180814-15 | *R.caudatum* | *Daphnia* |
| A3 | 10mL | 10 | 20180814-15 | *R.caudatum* | *Daphnia* |
| A4 | 10mL | 10 | 20180814-15 | *R.caudatum* | *Daphnia* |
| A5 | 10mL | 10 | 20180814-15 | *R.caudatum* | *Daphnia* |
| A6 | 10mL | 10 | 20180814-15 | *R.caudatum* | *Daphnia* |
| A1 | 100mL | 11 | 20180911-12 | *Vorticella* | *Eudiaptomus* |
| A2 | 100mL | 11 | 20180911-12 | *Vorticella* | *Eudiaptomus* |
| A3 | 100mL | 11 | 20180911-12 | *Vorticella* | *Eudiaptomus* |
| A4 | 100mL | 11 | 20180911-12 | *Vorticella* | *Eudiaptomus* |
| A5 | 100mL | 11 | 20180911-12 | *Vorticella* | *Eudiaptomus* |
| A6 | 100mL | 11 | 20180911-12 | *Vorticella* | *Eudiaptomus* |
| A1 | 10mL | 11 | 20180911-12 | *Histiobalantium* | *Eudiaptomus* |
| A2 | 10mL | 11 | 20180911-12 | *Histiobalantium* | *Eudiaptomus* |
| A3 | 10mL | 11 | 20180911-12 | *Histiobalantium* | *Eudiaptomus* |
| A4 | 10mL | 11 | 20180911-12 | *Histiobalantium* | *Eudiaptomus* |
| A5 | 10mL | 11 | 20180911-12 | *Histiobalantium* | *Eudiaptomus* |
| A6 | 10mL | 11 | 20180911-12 | *Histiobalantium* | *Eudiaptomus* |
| A1 | 10mL | 12 | 20181002-03 | *Vorticella* | *Cyclops* |
| A2 | 10mL | 12 | 20181002-03 | *Vorticella* | *Cyclops* |
| A3 | 10mL | 12 | 20181002-03 | *Vorticella* | *Cyclops* |
| A4 | 10mL | 12 | 20181002-03 | *Vorticella* | *Cyclops* |
| A5 | 10mL | 12 | 20181002-03 | *Vorticella* | *Cyclops* |
| A6 | 10mL | 12 | 20181002-03 | *Vorticella* | *Cyclops* |
| A1 | 200mL | 13 | 20181030-31 | *Vorticella* | *Eudiaptomus* |
| A2 | 200mL | 13 | 20181030-31 | *Vorticella* | *Eudiaptomus* |
| A3 | 200mL | 13 | 20181030-31 | *Vorticella* | *Eudiaptomus* |
| A4 | 200mL | 13 | 20181030-31 | *Vorticella* | *Eudiaptomus* |
| A5 | 200mL | 13 | 20181030-31 | *Vorticella* | *Eudiaptomus* |
| A6 | 200mL | 13 | 20181030-31 | *Vorticella* | *Eudiaptomus* |
| A1 | 10mL | 13 | 20181030-31 | *R.caudatum* | *Eudiaptomus* |
| A2 | 10mL | 13 | 20181030-31 | *R.caudatum* | *Eudiaptomus* |
| A3 | 10mL | 13 | 20181030-31 | *R.caudatum* | *Eudiaptomus* |
| A4 | 10mL | 13 | 20181030-31 | *R.caudatum* | *Eudiaptomus* |
| A5 | 10mL | 13 | 20181030-31 | *R.caudatum* | *Eudiaptomus* |
| A6 | 10mL | 13 | 20181030-31 | *R.caudatum* | *Eudiaptomus* |
| A1 | 100mL | 14 | 20181016-17 | *Vorticella* | *Cyclops* |
| A2 | 100mL | 14 | 20181016-17 | *Vorticella* | *Cyclops* |
| A3 | 100mL | 14 | 20181016-17 | *Vorticella* | *Cyclops* |
| A4 | 100mL | 14 | 20181016-17 | *Vorticella* | *Cyclops* |
| A5 | 100mL | 14 | 20181016-17 | *Vorticella* | *Cyclops* |
| A6 | 100mL | 14 | 20181016-17 | *Vorticella* | *Cyclops* |
| A1 | 10mL | 14 | 20181016-17 | *R.caudatum* | *Cyclops* |
| A2 | 10mL | 14 | 20181016-17 | *R.caudatum* | *Cyclops* |
| A3 | 10mL | 14 | 20181016-17 | *R.caudatum* | *Cyclops* |
| A4 | 10mL | 14 | 20181016-17 | *R.caudatum* | *Cyclops* |
| A5 | 10mL | 14 | 20181016-17 | *R.caudatum* | *Cyclops* |
| A6 | 10mL | 14 | 20181016-17 | *R.caudatum* | *Cyclops* |
| A1 | 200mL | 15 | 20181023-24 | *Vorticella* | *Cyclops* |
| A2 | 200mL | 15 | 20181023-24 | *Vorticella* | *Cyclops* |
| A3 | 200mL | 15 | 20181023-24 | *Vorticella* | *Cyclops* |
| A4 | 200mL | 15 | 20181023-24 | *Vorticella* | *Cyclops* |
| A5 | 200mL | 15 | 20181023-24 | *Vorticella* | *Cyclops* |
| A6 | 200mL | 15 | 20181023-24 | *Vorticella* | *Cyclops* |
| A1 | 10mL | 15 | 20181023-24 | *R.lacustris* | *Cyclops* |
| A2 | 10mL | 15 | 20181023-24 | *R.lacustris* | *Cyclops* |
| A3 | 10mL | 15 | 20181023-24 | *R.lacustris* | *Cyclops* |
| A4 | 10mL | 15 | 20181023-24 | *R.lacustris* | *Cyclops* |
| A5 | 10mL | 15 | 20181023-24 | *R.lacustris* | *Cyclops* |
| A6 | 10mL | 15 | 20181023-24 | *R.lacustris* | *Cyclops* |
| A1 | 10mL | 16 | 20180731-0801 | *Histiobalantium* | *Daphnia* |
| A2 | 10mL | 16 | 20180731-0801 | *Histiobalantium* | *Daphnia* |
| A3 | 10mL | 16 | 20180731-0801 | *Histiobalantium* | *Daphnia* |
| A4 | 10mL | 16 | 20180731-0801 | *Histiobalantium* | *Daphnia* |
| A5 | 10mL | 16 | 20180731-0801 | *Histiobalantium* | *Daphnia* |
| A6 | 10mL | 16 | 20180731-0801 | *Histiobalantium* | *Daphnia* |
| A1 | 100mL | 17 | 20180807-08 | *Histiobalantium* | *Daphnia* |
| A2 | 100mL | 17 | 20180807-08 | *Histiobalantium* | *Daphnia* |
| A3 | 100mL | 17 | 20180807-08 | *Histiobalantium* | *Daphnia* |
| A4 | 100mL | 17 | 20180807-08 | *Histiobalantium* | *Daphnia* |
| A5 | 100mL | 17 | 20180807-08 | *Histiobalantium* | *Daphnia* |
| A6 | 100mL | 17 | 20180807-08 | *Histiobalantium* | *Daphnia* |
| A1 | 200mL | 18 | 20180904-05 | *Histiobalantium* | *Daphnia* |
| A2 | 200mL | 18 | 20180904-05 | *Histiobalantium* | *Daphnia* |
| A3 | 200mL | 18 | 20180904-05 | *Histiobalantium* | *Daphnia* |
| A4 | 200mL | 18 | 20180904-05 | *Histiobalantium* | *Daphnia* |
| A5 | 200mL | 18 | 20180904-05 | *Histiobalantium* | *Daphnia* |
| A6 | 200mL | 18 | 20180904-05 | *Histiobalantium* | *Daphnia* |
| A1 | 200mL | 18 | 20180904-05 | *R.caudatum* | *Daphnia* |
| A2 | 200mL | 18 | 20180904-05 | *R.caudatum* | *Daphnia* |
| A3 | 200mL | 18 | 20180904-05 | *R.caudatum* | *Daphnia* |
| A4 | 200mL | 18 | 20180904-05 | *R.caudatum* | *Daphnia* |
| A5 | 200mL | 18 | 20180904-05 | *R.caudatum* | *Daphnia* |
| A6 | 200mL | 18 | 20180904-05 | *R.caudatum* | *Daphnia* |
| A1 | 100mL | 19 | 20181113-14 | *Histiobalantium* | *Eudiaptomus* |
| A2 | 100mL | 19 | 20181113-14 | *Histiobalantium* | *Eudiaptomus* |
| A3 | 100mL | 19 | 20181113-14 | *Histiobalantium* | *Eudiaptomus* |
| A4 | 100mL | 19 | 20181113-14 | *Histiobalantium* | *Eudiaptomus* |
| A5 | 100mL | 19 | 20181113-14 | *Histiobalantium* | *Eudiaptomus* |
| A6 | 100mL | 19 | 20181113-14 | *Histiobalantium* | *Eudiaptomus* |
| A1 | 200mL | 19 | 20181113-14 | *Histiobalantium* | *Eudiaptomus* |
| A2 | 200mL | 19 | 20181113-14 | *Histiobalantium* | *Eudiaptomus* |
| A3 | 200mL | 19 | 20181113-14 | *Histiobalantium* | *Eudiaptomus* |
| A4 | 200mL | 19 | 20181113-14 | *Histiobalantium* | *Eudiaptomus* |
| A5 | 200mL | 19 | 20181113-14 | *Histiobalantium* | *Eudiaptomus* |
| A6 | 200mL | 19 | 20181113-14 | *Histiobalantium* | *Eudiaptomus* |
| A1 | 10mL | 20 | 20180919-20 | *Histiobalantium* | *Cyclops* |
| A2 | 10mL | 20 | 20180919-20 | *Histiobalantium* | *Cyclops* |
| A3 | 10mL | 20 | 20180919-20 | *Histiobalantium* | *Cyclops* |
| A4 | 10mL | 20 | 20180919-20 | *Histiobalantium* | *Cyclops* |
| A5 | 10mL | 20 | 20180919-20 | *Histiobalantium* | *Cyclops* |
| A6 | 10mL | 20 | 20180919-20 | *Histiobalantium* | *Cyclops* |
| A1 | 100mL | 20 | 20180919-20 | *Histiobalantium* | *Cyclops* |
| A2 | 100mL | 20 | 20180919-20 | *Histiobalantium* | *Cyclops* |
| A3 | 100mL | 20 | 20180919-20 | *Histiobalantium* | *Cyclops* |
| A4 | 100mL | 20 | 20180919-20 | *Histiobalantium* | *Cyclops* |
| A5 | 100mL | 20 | 20180919-20 | *Histiobalantium* | *Cyclops* |
| A6 | 100mL | 20 | 20180919-20 | *Histiobalantium* | *Cyclops* |
| A1 | 200mL | 21 | 20180924-25 | *Histiobalantium* | *Cyclops* |
| A2 | 200mL | 21 | 20180924-25 | *Histiobalantium* | *Cyclops* |
| A3 | 200mL | 21 | 20180924-25 | *Histiobalantium* | *Cyclops* |
| A4 | 200mL | 21 | 20180924-25 | *Histiobalantium* | *Cyclops* |
| A5 | 200mL | 21 | 20180924-25 | *Histiobalantium* | *Cyclops* |
| A6 | 200mL | 21 | 20180924-25 | *Histiobalantium* | *Cyclops* |
| A1 | 100mL | 22 | 20181127-28 | *R.caudatum* | *Eudiaptomus* |
| A2 | 100mL | 22 | 20181127-28 | *R.caudatum* | *Eudiaptomus* |
| A3 | 100mL | 22 | 20181127-28 | *R.caudatum* | *Eudiaptomus* |
| A4 | 100mL | 22 | 20181127-28 | *R.caudatum* | *Eudiaptomus* |
| A5 | 100mL | 22 | 20181127-28 | *R.caudatum* | *Eudiaptomus* |
| A6 | 100mL | 22 | 20181127-28 | *R.caudatum* | *Eudiaptomus* |
| A1 | 200mL | 22 | 20181127-28 | *R.caudatum* | *Eudiaptomus* |
| A2 | 200mL | 22 | 20181127-28 | *R.caudatum* | *Eudiaptomus* |
| A3 | 200mL | 22 | 20181127-28 | *R.caudatum* | *Eudiaptomus* |
| A4 | 200mL | 22 | 20181127-28 | *R.caudatum* | *Eudiaptomus* |
| A5 | 200mL | 22 | 20181127-28 | *R.caudatum* | *Eudiaptomus* |
| A6 | 200mL | 22 | 20181127-28 | *R.caudatum* | *Eudiaptomus* |
| A1 | 100mL | 23 | 20181120-21 | *R.caudatum* | *Cyclops* |
| A2 | 100mL | 23 | 20181120-21 | *R.caudatum* | *Cyclops* |
| A3 | 100mL | 23 | 20181120-21 | *R.caudatum* | *Cyclops* |
| A4 | 100mL | 23 | 20181120-21 | *R.caudatum* | *Cyclops* |
| A5 | 100mL | 23 | 20181120-21 | *R.caudatum* | *Cyclops* |
| A6 | 100mL | 23 | 20181120-21 | *R.caudatum* | *Cyclops* |
| A1 | 200mL | 23 | 20181120-21 | *R.caudatum* | *Cyclops* |
| A2 | 200mL | 23 | 20181120-21 | *R.caudatum* | *Cyclops* |
| A3 | 200mL | 23 | 20181120-21 | *R.caudatum* | *Cyclops* |
| A4 | 200mL | 23 | 20181120-21 | *R.caudatum* | *Cyclops* |
| A5 | 200mL | 23 | 20181120-21 | *R.caudatum* | *Cyclops* |
| A6 | 200mL | 23 | 20181120-21 | *R.caudatum* | *Cyclops* |
| A1 | 100mL | 24 | 20180724-25 | *R.lacustris* | *Daphnia* |
| A2 | 100mL | 24 | 20180724-25 | *R.lacustris* | *Daphnia* |
| A3 | 100mL | 24 | 20180724-25 | *R.lacustris* | *Daphnia* |
| A4 | 100mL | 24 | 20180724-25 | *R.lacustris* | *Daphnia* |
| A5 | 100mL | 24 | 20180724-25 | *R.lacustris* | *Daphnia* |
| A6 | 100mL | 24 | 20180724-25 | *R.lacustris* | *Daphnia* |
| A1 | 200mL | 24 | 20180830-0901 | *R.lacustris* | *Daphnia* |
| A2 | 200mL | 25 | 20180830-0901 | *R.lacustris* | *Daphnia* |
| A3 | 200mL | 25 | 20180830-0901 | *R.lacustris* | *Daphnia* |
| A4 | 200mL | 25 | 20180830-0901 | *R.lacustris* | *Daphnia* |
| A5 | 200mL | 25 | 20180830-0901 | *R.lacustris* | *Daphnia* |
| A6 | 200mL | 25 | 20180830-0901 | *R.lacustris* | *Daphnia* |
| A1 | 10mL | 25 | 20181024-25 | *R.lacustris* | *Eudiaptomus* |
| A2 | 10mL | 26 | 20181024-25 | *R.lacustris* | *Eudiaptomus* |
| A3 | 10mL | 26 | 20181024-25 | *R.lacustris* | *Eudiaptomus* |
| A4 | 10mL | 26 | 20181024-25 | *R.lacustris* | *Eudiaptomus* |
| A5 | 10mL | 26 | 20181024-25 | *R.lacustris* | *Eudiaptomus* |
| A6 | 10mL | 26 | 20181024-25 | *R.lacustris* | *Eudiaptomus* |
| A1 | 100mL | 27 | 20181204-05 | *R.lacustris* | *Eudiaptomus* |
| A2 | 100mL | 27 | 20181204-05 | *R.lacustris* | *Eudiaptomus* |
| A3 | 100mL | 27 | 20181204-05 | *R.lacustris* | *Eudiaptomus* |
| A4 | 100mL | 27 | 20181204-05 | *R.lacustris* | *Eudiaptomus* |
| A5 | 100mL | 27 | 20181204-05 | *R.lacustris* | *Eudiaptomus* |
| A6 | 100mL | 27 | 20181204-05 | *R.lacustris* | *Eudiaptomus* |
| A1 | 200mL | 27 | 20181204-05 | *R.lacustris* | *Eudiaptomus* |
| A2 | 200mL | 27 | 20181204-05 | *R.lacustris* | *Eudiaptomus* |
| A3 | 200mL | 27 | 20181204-05 | *R.lacustris* | *Eudiaptomus* |
| A4 | 200mL | 27 | 20181204-05 | *R.lacustris* | *Eudiaptomus* |
| A5 | 200mL | 27 | 20181204-05 | *R.lacustris* | *Eudiaptomus* |
| A6 | 200mL | 27 | 20181204-05 | *R.lacustris* | *Eudiaptomus* |
| A1 | 100mL | 28 | 20181106-07 | *R.lacustris* | *Cyclops* |
| A2 | 100mL | 28 | 20181106-07 | *R.lacustris* | *Cyclops* |
| A3 | 100mL | 28 | 20181106-07 | *R.lacustris* | *Cyclops* |
| A4 | 100mL | 28 | 20181106-07 | *R.lacustris* | *Cyclops* |
| A5 | 100mL | 28 | 20181106-07 | *R.lacustris* | *Cyclops* |
| A6 | 100mL | 28 | 20181106-07 | *R.lacustris* | *Cyclops* |
| A1 | 200mL | 28 | 20181106-07 | *R.lacustris* | *Cyclops* |
| A2 | 200mL | 28 | 20181106-07 | *R.lacustris* | *Cyclops* |
| A3 | 200mL | 28 | 20181106-07 | *R.lacustris* | *Cyclops* |
| A4 | 200mL | 28 | 20181106-07 | *R.lacustris* | *Cyclops* |
| A5 | 200mL | 28 | 20181106-07 | *R.lacustris* | *Cyclops* |
| A6 | 200mL | 28 | 20181106-07 | *R.lacustris* | *Cyclops* |
